# Free Margin Running Suture Repair for Bileaflet Mitral Valve Prolapse in Patients with Left Ventricular Dysfunction: A Mid-term Follow-up Study

**DOI:** 10.1093/icvts/ivaf187

**Published:** 2025-08-09

**Authors:** Alfonso Agnino, Antonio D’Errico Ramirez, Matteo Parrinello, Salvatore Muccio, Amedeo Anselmi, Vito Giovanni Ruggieri

**Affiliations:** Division of Robotic and Minimally Invasive Cardiac Surgery, HUMANITAS Gavazzeni-Castelli, Bergamo, 24125, Italy; Division of Thoracic and Cardiovascular Surgery, CHU Reims, Rue du General Koenig, Reims, 51100, France; Division of Robotic and Minimally Invasive Cardiac Surgery, HUMANITAS Gavazzeni-Castelli, Bergamo, 24125, Italy; Division of Thoracic and Cardiovascular Surgery, CHU Reims, Rue du General Koenig, Reims, 51100, France; Division of Thoracic and Cardiovascular Surgery, Pontchaillou University Hospital, Rennes, 35000, France; Division of Thoracic and Cardiovascular Surgery, CHU Reims, Rue du General Koenig, Reims, 51100, France; URCA, Université de Reims Champagne Ardenne, Reims, 51100, France

**Keywords:** free margin running suture technique, mitral valve repair, robotic mitral valve repair, left ventricular dysfunction

## Abstract

**Objectives:**

Repairing severe mitral regurgitation (MR) in patients with degenerative bileaflet prolapse and reduced left ventricular ejection fraction (LVrEF) is challenging. The Free Margin Running Suture (FMRS) technique offers a non-resectional approach, but mid-term data are limited.

**Methods:**

We analysed 28 patients with bileaflet degenerative MR and LVrEF (≤40%) undergoing FMRS. Primary outcomes were mid-term survival and MR recurrence; secondary outcomes included LVEF, in-hospital complications, transmitral gradient, coaptation length, and mitral valve orifice area.

**Results:**

Mean age was 59.3 ± 12.8 years; 69.4% were male. No perioperative deaths; 1 patient required ECMO. Mean aortic cross-clamp time was 47 ± 18.6 min. Over 4.7 years, survival was 100%, with 1 case of moderate MR recurrence. At follow-up, LVEF improved to 43.04 ± 2.26 (*P* < 0.001), and all patients were NYHA I-II.

**Conclusions:**

FMRS is a minimally invasive, reproducible technique providing durable repair, symptomatic improvement, and excellent mid-term survival.

## Graphical Abstract

**Figure:**
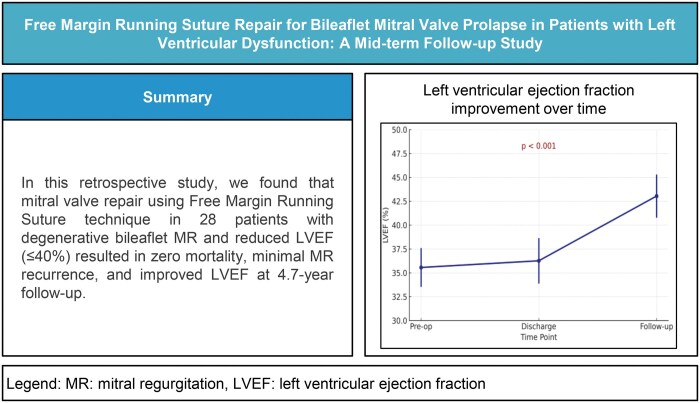

## INTRODUCTION

Mitral valve (MV) repair is the surgical intervention of first choice in degenerative primary mitral regurgitation (MR).^1–4^ MR in patients with reduced left ventricular ejection fraction (LVrEF) and advanced heart failure symptoms remains a significant clinical challenge.^5–7^ Surgical MV repair offers better outcomes compared to valve replacement, including preserved ventricular function and reduced long-term mortality.^8–12^ However, bileaflet prolapse with eccentric regurgitant jets, particularly in the context of LVrEF, poses technical challenges for durable repair. In current real-world practice, this subset of high-surgical-risk patients is treated with MV transcatheter edge-to-edge repair (M-TEER). However, limitations such as inadequate MR reduction, leaflet injury, mitral stenosis, and short follow-up durations still remain.^13^ The Free Margin Running Suture (FMRS) technique first described by Agnino in 2017 represents an evolution in MV repair strategies, offering a non-resectional approach aimed at preserving native leaflet motion and subvalvular anatomy.^14–17^ Although the technique has demonstrated efficacy in cases of posterior leaflet prolapse, its application in complex bileaflet disease and LVrEF patients was not previously reported. This study evaluates the mid-term outcomes of FMRS in patients with degenerative multi-segment bileaflet disease, severe MR and LVrEF, focusing on its impact on survival, symptoms improvements, valve performance, and left ventricular function.

## METHODS

### Patient selection

From February 2011 to October 2019, 136 consecutive patients with degenerative disease underwent FMRS MV repair in our department. This retrospective study was conducted in accordance with the ethical principles outlined in the Declaration of Helsinki and the Declaration of Taipei (World Medical Association, 2016), particularly concerning the responsible use of health databases and biobanks. Data used in this study were anonymized prior to analysis to ensure the protection of individual privacy and comply with ethical standards for secondary use of data. This study was reviewed and approved by the Institutional Review Board of HUMANITAS Gavazzeni-Castelli, Bergamo, Italy, protocol number 7/24 GAV. Exclusion criteria included ischaemic MR, prior mitral surgery, LVEF > 40%, isolated leaflet prolapse, chordae rupture, mitral annular calcification, and endocarditis. Twenty-eight consecutive patients with severe degenerative MR secondary to bileaflet prolapse and reduced LVEF (≤40%) were encompassed. All patients exhibited eccentric regurgitant jets indicating non-balanced bileaflet prolapse.

### Cardiac imaging

Each patient received a detailed preoperative and intraoperative multiplane transoesophageal echocardiographic evaluation, focusing on MV and annular structure, specific segmental abnormalities, and the condition of the subvalvular apparatus. Particular attention was given to analysing the characteristics of the regurgitant jet. Eligibility for FMRS was limited to patients presenting with non-balanced MV prolapse, defined by prolapse of both leaflets—predominantly the posterior leaflet—resulting in an eccentric jet directed towards the interatrial septum.^18^ Every patients also underwent low-dose dobutamine stress echocardiography (5-20 µg/kg/min) to assess LV contractile reserve. Increase in stroke volume or in LVEF ≥ 5%-10% suggested presence of myocardial viability.^19^

### Surgical procedure

All surgical approaches were considered (median sternotomy, video-assisted right minithoracotomy with or without aortic endo-clamping and robotic-assisted surgery). In general, the choice of surgical approach was based on personal experience and the introduction of new surgical techniques. Our surgical series spans from February 2011 to November 2019. At our centre, we introduced right mini-thoracotomy for mitral repair in 2012 and robotic surgery in early 2018. Patients with complex heart disease were approached with minimally invasive techniques only once we had gained sufficient expertise and experience with simpler cases. Procedures were performed by 3 surgeons (A.A., A.A., R.G.V.) with the patients under mildly hypothermic cardiopulmonary bypass (CPB). The FMRS technique was performed as previously described, using a double-armed 4-0 expanded polytetrafluoroethylene (PTFE) suture. The suture was passed through the free margin of prolapsing segments to constrain leaflet height, creating a coaptation surface (see **Supplementary Material—Video S1**). Complete annuloplasty was performed in all cases using appropriately sized semi-rigid annuloplasty rings.

### Follow-up

Operative mortality and postoperative complications were defined as events occurring within 30 days after surgery or later if they occurred during the same hospital stay. Early complications were categorized into MV-related, non-mitral cardiac, or extracardiac events, following the classification system proposed by Akins et al.^20^ Discharge echocardiographic assessments included measurements of residual MR, mean transvalvular mitral gradient, left ventricular ejection fraction, as well as left ventricular systolic and end-diastolic diameters and volumes. MR severity was graded as follows: mild (1+/4+), defined by an effective regurgitant orifice area (EROA) <20 mm^2^ and/or a regurgitant volume (RVol) <30 mL; mild-to-moderate (2+/4+), with an EROA of 20-29 mm^2^ and RVol of 30-39 mL; moderate (3+/4+), defined by an EROA of 30-44 mm^2^ and RVol of 45-59 mL; and severe (4+/4+), characterized by an EROA ≥40 mm^2^ and/or RVol ≥60 mL. Both preoperative and postoperative echocardiograms were conducted in accordance with current clinical guidelines by board-certified cardiologists. All patients were followed up in late 2024 via outpatient evaluations and/or communication with their referring cardiologist. At least 1 follow-up resting echocardiogram, interpreted by a board-certified cardiologist, was obtained for each patient. The final clinical and echocardiographic assessment date was used to determine the last follow-up point. Additionally, we report outcomes from the 4.7-year follow-up interval. MR was classified as residual if present at hospital discharge, or recurrent if it developed later in patients with initially satisfactory discharge imaging. For end-point determination, both residual and recurrent MR were considered clinically significant if graded at 2+/4+ (mild to moderate) or greater.

### Statistical analysis

The study design was retrospective. All data were collected and entered into a dedicated electronic database. Follow-up was 100% complete, and no patients were lost to follow-up, ensuring the integrity of the dataset. Continuous variables were summarized as means and standard deviations, calculated directly from the available complete dataset. For comparisons between paired measurements (eg, discharge vs follow-up values), appropriate paired statistical tests were used. Prior to hypothesis testing, the normality of each variable’s distribution was assessed using standard statistical methods (Shapiro-Wilk test). Based on the distribution characteristics: if the data were normally distributed, a paired Student’s t-test was applied; if the data did not meet the criteria for normality, the Wilcoxon signed-rank test was used as a non-parametric alternative. All statistical analyses were conducted using standard programming environments suitable for biomedical research, such as MATLAB or R. The threshold for statistical significance was set at α = 0.05. All reported p-values were 2-tailed and unadjusted for multiplicity.

## RESULTS

### Baseline and intraoperative characteristics

The mean age of the study population was 59.3 ± 12.8 years, with 75% being male. All patients had a LVrEF of 35.56 ± 2.03% and were symptomatic for dyspnoea, experiencing significant limitations in daily activities (NYHA functional class III-IV: 100%). No patients were obese, with a mean body mass index (BMI) of 23.3 ± 2.9 kg/m^2^. Two patients (7.1%) had undergone previous non-mitral cardiac surgery. Moderate pulmonary hypertension was present in all patients, with a pulmonary artery systolic pressure (PASP) of 40.1 ± 8.3 mmHg, while right ventricular function remained normal (TAPSE: 18 ± 1.5 mm). No patient had more-than-moderate tricuspid regurgitation or significant tricuspid anular dilatation. Bileaflet MV disease was observed in 100% of patients. Minimally invasive surgery was performed using a robot-assisted technique in 3 patients (10.7%), a right mini-thoracotomy in 8 patients (28.5.2%), while 17 patients (60.7%) underwent surgery via median sternotomy. Annuloplasty was performed using a complete ring in all 28 patients (100%). Large prosthetic ring sizes were utilized, including 38 mm in 2 patients (7.1%), 40 mm in 10 patients (35.7%), and 42 mm in 16 patients (57.1%). The mean aortic cross-clamp time was 47 ± 18.6 min. All patients left the operating room with no more than trivial MR, as confirmed by off-pump intraoperative transoesophageal echocardiography. There were no perioperative mortalities. One patient (3.5%) required postoperative ECMO for postcardiotomy shock. No patients experienced systolic anterior motion (SAM) or significant mitral stenosis post-repair. Baseline, echocardiographic, and operative data are summarized in **Tables 1 and 2**.

**Table 1.** Baseline Characteristics of the Population (*N* = 28)

Characteristics	*N* (%) or average ± SD
Age (years)	59.3 ± 12.8
Male gender	75 (69.4)
Body mass index (kg/m²)	23.3 ± 2.9
NYHA functional class III or IV	28 (100)
Previous cardiac surgery	2 (7.1)
LVEF (%)	35.56 ± 2.03
LVEDD (mm)	71.65 ± 3.03
LVESD (mm)	54.25 ± 3.6
LVEDV (mL)	153.3 ± 7.68
LVESV (mL)	91.9 ± 7.22
PASP (mmHg)	40.1 ± 8.3
TAPSE	18 ± 1.5

Abbreviations: LVEDD: left ventricular end-diastolic diameter; LVEDV: left ventricular end-diastolic volume; LVEF: left ventricular ejection fraction; LVESD: left ventricular end-systolic diameter; LVESV: left ventricular end-systolic volume; NYHA: New York Heart Association; PASP: pulmonary artery systolic pressure; SD: standard deviation; TAPSE: tricuspid annular plane systolic excursion.

**Table 2.** Intraoperative Characteristics and Early Postoperative Results in the Overall Population (*N* = 28)

Surgical approach	
Minimally invasive (right minithoracotomy)	8 (28.5)
Robot-assisted	3 (10.7)
Median sternotomy	17 (60.7)
Bileaflet disease	28 (100)
Prosthetic ring size (mm)	
38	2 (7.14)
40	10 (35.71)
42	16 (57.14)
CPB time (min)	68.4 ± 25.6
Aortic cross-clamp time (min)	47 ± 18.6
ICU stay (h)	86.9 ± 11.6
Mechanical ventilation time (h)	32.34 ± 5.7
Blood product transfusions	19 (67.8)
ECMO	1 (3.5)

Abbreviations: CPB: cardiopulmonary bypass; ECMO: extracorporeal membrane oxygenation; ICU: intensive care unit; SD: standard deviation.

### Early postoperative results

No postoperative mortality was observed. One patient (3.5%) required extracorporeal life support (ECLS) for post-cardiotomy shock, and 1 patient who underwent FMRS repair developed postcardiotomy shock due to left ventricular failure and was treated with femoro-femoral VA-ECMO. Once haemodynamically stabilized, the patient underwent diagnostic coronary angiography, which was negative for injury to the circumflex artery. Cardiocirculatory support was maintained for 2 days. The total hospital stay was 18 days, after which the patient was transferred to a cardiopulmonary rehabilitation facility. The mean intensive care unit (ICU) stay was 86.9 ± 11.6 hours, while the mean mechanical ventilation time was 32.3 ± 5.7 hours. Blood product transfusion was required in 19 patients (67.8%). Echocardiographic findings confirmed excellent MV repair outcomes, with no residual MR in 21 patients (75%) and trivial MR in 7 patients (25%). None of the patients had mild to moderate MR. The mean left ventricular ejection fraction (LVEF) at discharge was 36.2% ± 2.3%. The average transmitral gradient was 4.3 ± 1.4 mmHg, the mean coaptation length was 1.1 ± 0.3 cm, and the MV orifice area was 3.5 ± 0.6 cm^2^.

Early postoperative data are summarized in **Table 2**.

### Mid-term outcomes

The mean clinical and echocardiographic follow-up was 4.7 years. No patients died during follow-up. At the last evaluation, 100% of patients were in NYHA functional class I-II, indicating sustained symptomatic improvement. Mid-term echocardiographic follow-up confirmed excellent durability of MV repair. No residual MR was observed in 20 patients (71.4%), while 8 patients (28.5%) had trivial MR. No patients experienced severe MR or required reoperation. According to the results obtained, all variables were statistically significant except for coaptation length. LVEF significantly improved over time, increasing from 36.26 ± 2.38 at discharge to 43.04 ± 2.26 (<0.001) at follow-up. Also the left ventricular size decreased over time with important statistical significance at discharge from follow-up. Comparison of echocardiographic data at discharge and at 4.7 years follow-up demonstrated excellent repair durability:

- No significant transvalvular gradient was detected (4.5 ± 1.4 mmHg vs 3.4 ± 0.8 mmHg, *P* < 0.001).
- An effective and large mitral orifice area was maintained (3.5 ± 0.6 cm^2^ vs 3.9 ± 0.8 cm^2^, *P* = 0.001).
- Coaptation length remained stable over time (1.1 ± 0.3 cm vs 1.1 ± 0.1 cm, *P* = 0.9).

Echocardiographic findings at discharge and at the 4.7-year follow-up examination are given in **Table 3**.

**Table 3.** Echocardiographic Findings at Discharge and at the 4.7-Year Follow-up Examination (*N* = 28)

Characteristics	At discharge	At follow-up		*P*-value
Degree of mitral regurgitation, *n* (%)				0.05
None	21 (75)	20 (71.4)		
Trivial (1+/4+)	7 (25)	7 (25)		
Average transmitral gradient (mmHg), mean ± SD	4.3 ± 1.4	3.4 ± 0.8		<0.001
LVEF (%), mean ± SD	36.26 ± 2.38	43.04 ± 2.26		<0.001
LVEDD (mm), mean ± SD	69.25 ± 2.38	61.40 ± 2.91		<0.001
LVESD (mm), mean ± SD	53.05 ± 3.63	49.35 ± 3.20		0.001
LVEDV (mL), mean ± SD	1144.45 ± 2.38	122.15 ± 8.01		<0.001
LVESV (mL), mean ± SD	89.35 ± 5.76	70.75 ± 6.29		<0.001
Coaptation length (cm), mean ± SD	1.1 ± 0.3	1.1 ± 0.1		0.9
Mitral valve orifice area (cm²), mean ± SD	3.5 ± 0.6	3.9 ± 0.8		0.001

Abbreviations: LVEDD: left ventricular end-diastolic diameter; LVEDV: left ventricular end-diastolic volume; LVEF: left ventricular ejection fraction; LVESD: left ventricular end-systolic diameter; LVESV: left ventricular end-systolic volume; SD: standard deviation.

Patients with tricuspid regurgitation did not exhibit significant annular dilatation and were therefore not treated surgically. The degree of tricuspid regurgitation was not specifically analysed during follow-up data collection. However, none of the 28 patients in the series underwent tricuspid valve surgery during follow-up, nor did they develop clinical signs of right heart failure.

## DISCUSSION

The FMRS has been already demonstrated effective in “generic” primary MR, anatomically elegible patients.^14^^,^^15^ In this report, we propose this technique as a tailored approach for a specific and challenging subpopulation, providing a novel solution to complex bileaflet MV prolapse in the setting of reduced LVEF. The technique preserves leaflet architecture and mobility, which is critical in maintaining ventricular dynamics.^21^ The avoidance of leaflet resection reduces the risk of restrictive pathologies, which are more likely to occur with traditional resection-based approaches.^22^

The clinical outcomes in this study highlight the durability and efficacy of FMRS in a high-risk population. Zero late mortality and no recurrence of significant MR underscore its long-term reliability.^23^ The echocardiographic findings are particularly noteworthy, with stable coaptation length and low transmitral gradients. These findings align with the principles of creating a robust coaptation zone and preserving mitral apparatus.

Compared to alternative techniques, FMRS simplifies the repair process and reduces operative times. In the present study, the mean aortic cross-clamp time was reported to be only 47 min, which would be considered an excellent outcome, particularly given the use of minimally invasive and robotic approaches. This value appears to be notably shorter than those reported in a recent meta-analysis involving 1905 patients who underwent minimally invasive cardiac surgery (MICS) or sternotomy for complex bileaflet MV repairs incorporating both resection and artificial chordal implantation. In that analysis, patients in the MICS group were found to have experienced significantly longer CPB and cross clamp time (XCT) times compared to those in the sternotomy group (CPB: 129.2 vs 97.0 min; ACC: 85.6 vs 63.4 min, respectively).^24^ Enhanced procedural efficiency could be particularly beneficial in patients with LVrEF, for whom prolonged CPB and ischaemic times are associated with poorer postoperative outcomes.^25–27^

In comparison with TEER, FMRS might also offer superior clinical durability. In the EVEREST II trial, TEER was associated with 3+/4+ residual MR in 17.9% of patients at 1-year follow-up, increasing to 18.8% at 5 years. Furthermore, the cumulative incidence of MV surgery or reoperation was reported to reach 27.9% at 5 years.^28^ Although newer-generation TEER systems may demonstrate improved procedural outcomes, published follow-up data remain limited to 1 year. In the recently reported CLASP IID trial, 2% of patients were found to exhibit recurrent moderate or severe MR at 6 months, and 1% of patients with degenerative MR reportedly required MV reintervention following TEER.^29^ Notably, when surgical intervention becomes necessary after TEER, most patients would likely undergo MV replacement rather than repair.^30^

### Biomechanical considerations

The FMRS technique entails the placement of a continuous 4-0 expanded PTFE suture along the free edge of all 3 posterior mitral leaflet segments. This approach anchors prolapsing segments to at least 1 anatomically normal, non-prolapsing segment (typically P1 or P3), effectively re-establishing alignment with the annular plane. By folding the prolapsing tissue and preserving the subvalvular apparatus, the technique enhances posterior leaflet coaptation height and shifts the coaptation zone anteriorly, thereby increasing the overall leaflet coaptation surface. These biomechanical changes are central to the mechanism of FMRS in addressing bileaflet prolapse. In such cases, anterior leaflet prolapse often arises from annular dilatation rather than intrinsic pathology; thus, the combined use of an annuloplasty ring and anterior displacement of the coaptation line typically suffices to restore anterior leaflet competence. Preoperative assessment of the anterior leaflet is essential, as the presence of ruptured chordae or substantial leaflet redundancy may contraindicate the use of FMRS alone. In our series, cases of bileaflet prolapse were successfully managed using the FMRS technique alone, without the need for additional leaflet procedures. This outcome suggests that the excess anterior leaflet tissue may be effectively constrained and redistributed across the coaptation surface, which is typically displaced anteriorly following FMRS. Although we are unable to assess whether anterior leaflet involvement is associated with late recurrence of MR, given that no recurrent regurgitation events occurred in our cohort, precluding multivariate analysis, it is plausible that the increased coaptation surface, combined with preservation of the posterior leaflet and subvalvular apparatus, facilitates a more uniform distribution of mechanical stress. This, in turn, may enhance the durability of the repair over the mid-term follow-up period.^14^ The FMRS technique appears particularly well-suited for complex cases involving multi-segment bileaflet prolapse, where nonresectional approaches are preferred to maintain native leaflet geometry and avoid distortion. Importantly, the absence of SAM observed in this cohort underscores the safety of the technique. This finding is likely attributable to the consistent use of larger annuloplasty rings, which help maintain favourable anterior-posterior annular dimensions and reduce the risk of SAM.

### Clinical implications

The findings of this study support FMRS as a viable and effective option for patients with complex degenerative MR and advanced heart failure. By addressing the anatomical challenges of bileaflet prolapse without compromising native valve dynamics, FMRS enhances both surgical outcomes and mid-term repair durability.

Importantly, this technique offers several practical advantages:

- It reduces inter-surgeon variability and reliance on subjective judgement by providing clear anatomical landmarks and a standardized suture strategy.
- It preserves physiological valve function and anatomy while promoting consistency in repair techniques.
- It simplifies the procedure, facilitating adoption by less experienced or junior surgeons.
- It allows for final adjustments, even after ring implantation, without significant effort or impaired exposure.^31^

### Limitations and future directions

While the results are promising, the study’s limitations include its small sample size and 2-centre design. The relatively short follow-up period, although longer than many initial reports, limits the ability to assess long-term durability. Future multicentre studies with larger cohorts and extended follow-up periods are needed to confirm these findings and establish FMRS as a gold standard for complex MV repair. In addition, comparative studies between FMRS and resection-based techniques would provide valuable insights into their relative advantages, particularly in patients with severe LV dysfunction.

## CONCLUSION

The FMRS technique represents a potential paradigm shift for the surgical management of degenerative bileaflet MV disease, particularly in patients with reduced LVEF. Its simplicity, safety, and excellent mid-term outcomes make it an attractive tool in the armamentarium of mitral surgeons.

## Supplementary Material

ivaf187_Supplementary_Data

## Data Availability

The datasets generated and analysed during this study are available from the corresponding author upon reasonable request.
