# Supplementary material for: Free Margin Running Suture Repair for Bileaflet Mitral Valve Prolapse in Patients with Left Ventricular Dysfunction: A Mid-term Follow-up Study
Source: Interdiscip Cardiovasc Thorac Surg. 2025 Aug 9;40(8):ivaf187. doi: 10.1093/icvts/ivaf187 (PMC12371330; doi:10.1093/icvts/ivaf187)
Supplement: ivaf187_Supplementary_Data [file ivaf187_supplementary_data.pdf]

Supplementary Material:
Video:

https://ctsnet.figshare.com/articles/media/Minimally_Invasive_Mitral_Valve_Repair_Through_Nonresectional_Posterior_Leaflet_Remodeling/7757261/1

Thanks to. A. Agnino et A.Anselmi for providing this FMRS Technique descriptive video.
